# A Review of HDV Infection

**DOI:** 10.3390/v14081749

**Published:** 2022-08-10

**Authors:** Gian Paolo Caviglia, Alessia Ciancio, Mario Rizzetto

**Affiliations:** 1 Department of Medical Sciences, University of Turin, 10126 Turin, Italy; 2 Unit of Gastroenterology, “Città della Salute e della Scienza di Torino” Molinette Hospital, 10126 Turin, Italy

**Keywords:** hepatitis D virus, hepatitis D, Kolmioviridae, anti-HD, bulevirtide

## Abstract

Hepatitis D is the most severe viral hepatitis. Hepatitis D virus (HDV) has a very small RNA genome with unique biological properties. It requires for infection the presence of hepatitis B virus (HBV) and is transmitted parenterally, mainly by superinfection of HBsAg carriers who then develop chronic hepatitis D. HDV has been brought under control in high-income countries by the implementation of HBV vaccination, and the clinical pattern has changed to a chronic hepatitis D seen in ageing patients with advanced fibrotic disease; the disease remains a major health concern in developing countries of Africa and Asia. Every HBsAg-positive subject should be tested for HDV serum markers by reflex testing, independently of clinical status. Vaccination against HBV provides the best prophylaxis against hepatitis D. The only therapy available so far has been the poorly performing Interferon alfa; however, several new and promising therapeutic approaches are under study.

## 1. Introduction

The clue that led to the discovery of hepatitis D virus (HDV) was the identification in the mid-1970 s in Torino of the delta antigen, a novel immunologic reactivity in subjects infected with hepatitis B virus (HBV) and severe liver disease. Given the invariable association with HBV, the delta antigen was first assumed to be an expression of HBV and was reported in 1977 as a likely variant of HBV [1]. The discovery raised the interest of the National Institute of Health, which made available in 1978 the chimpanzee model for the study of the novel antigen. In the United States, delta was retrieved in large amounts from the blood of hepatitis B surface antigen (HBsAg)-positive chimpanzees superinfected with the serum of an Italian patient exhibiting the antigen in liver, leading to the isolation of a 35 to 37 nm particle containing a 1.7 kb RNA, smaller in size than the HBV genome and of all known animal viruses [2]. The birth of hepatitis D virus was in 1980; it was originally classified as the only member of the genus Deltavirus (family Delatviridae) [3], and was recently reclassified together with other HDV-like viruses, as Kolmioviridae, the only family of the Ribozyviria; the term kolmio means “triangle” in Finnish, in reference to the Greek letter “Δ” (delta) [4].

This review discusses the progress in the understanding of the biological life cycle of HDV, the striking achievements on its virology and evolution, the contemporary epidemiologic scenario of the infection in Italy and the current therapeutic perspectives of hepatitis D.

## 2. The HDV as a Unique Human Pathogen

### 2.1. The Life Cycle of the HDV

**Figure 1:**
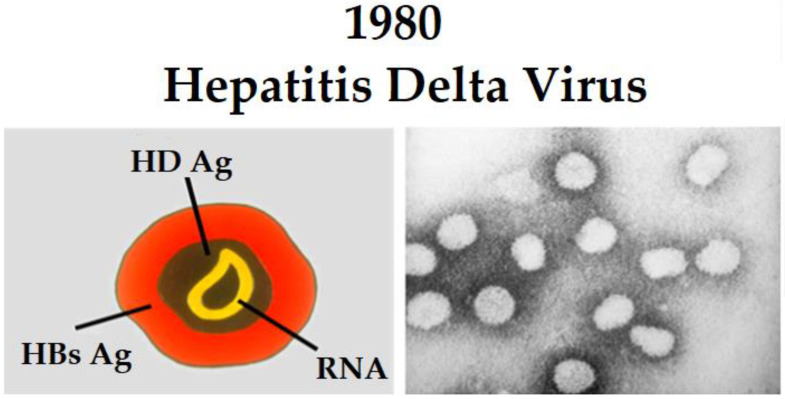
Hepatitis delta virus. Defective, dependent on biological help from the HBV, present only in HBsAg-positive subjects (HBsAg carriers). Virion: 36 nm particle, enveloped in HBsAg. Genome: 1.7 Kb RNA. Abbreviations: HBsAg, hepatitis B surface antigen; HDAg, hepatitis D antigen.

The HDV genome consists of a circular, single-stranded minus RNA of about 1700 nucleotides enclosed in a particle coated by HBsAg (HDV virion), with no nucleocapsid structure (Figure 1) [5]. The HDV-RNA folds into an unbranched rod-like structure in which the 70% of the nucleotides are paired. There are eight different HDV genotypes, with different geographic distribution; their lengths are restricted to a range of 1672 to 1697 nucleotides, with up to 35% divergence between genotypes and 81–89 percent homology in nucleotide sequences within the same genotype [6].

HDV enters into hepatocytes by attaching through the envelope protein HBsAg to the sodium taurocholate co-transporting polypeptide (NTCP). A rolling circle mechanism, common to plant viroids but unknown to animal viruses, leads in the nuclei to the replication of genomic RNA into the antigenome. With no need for an enzymatic activity, HDV-RNA can self-cleave both the genomic and antigenomic strands using a ribozyme fewer than 100 nucleotides long in the RNA molecule [7,8].

**Figure 2:**
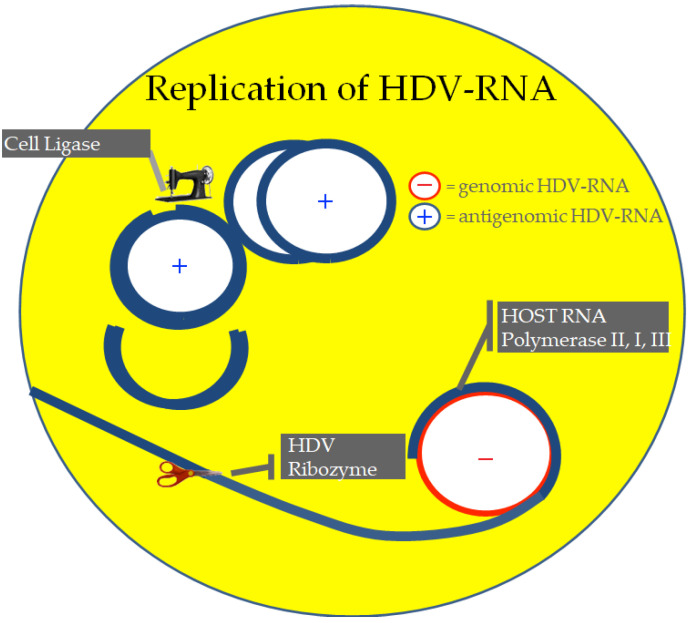
Replication of HDV-RNA. The HDV is replicated by DNA-dependent RNA polymerases of the hepatocytes, redirected to copy the viral RNA. The host RNA polymerases elongate a multimeric linear RNA transcript over the circular genome, which is then cut to monomeric linear HDV-RNA by the ribozyme and ultimately ligated into the circular infectious form by cell ligases.

The recognition that the viral genome is replicated by cellular DNA-dependent RNA polymerases, primarily by RNA polymerase II, redirected to use the rod-like viral RNA template as if it were a DNA template, provided the answer to the initial conundrum of how HDV-RNA could replicate in the absence of a viral RNA polymerase (Figure 2) [9].

The HD antigen (HD-Ag) is the only protein expressed by the HDV. Following a post-translational modification, two forms of HD-Ag are generated: the 24 kDa small HD-Ag (s-HD-Ag) and the 27 kDa large HD-Ag (l-HD-Ag) [10]. The original s-HD-Ag is 195 amino acids (aa) long, and is necessary for HDV-RNA replication and promotes viral RNA accumulation in cells. The l-HD-Ag is 19 aa longer with a final length of 214 aa; it acts by inhibiting HDV-RNA replication and is essential for the assembly and secretion of HD virions.

Both HD-Ag isoforms undergo many other post-translational changes that play key roles in modifying the HD-Ag functions and supply molecular switches that modulate HDV’s life cycle [11]; in particular, the farnesylation of l-HD-Ag by a host farnesyl transferase is necessary to drive the HDV-RNA to interact with HBsAg in virion assembly [12].

### 2.2. Replication of the HDV without the HBV

Though natural HDV infections occur only in individuals with HBsAg in blood, inside the hepatocytes, host RNA polymerases replicate HDV-RNA without the involvement of HBV [5]; therefore, mammalian cells can independently support effective intrahepatic HDV replication under experimental conditions. HDV replication was induced in cell cultures transfected with cDNA constructs of HDV and in several tissues of mice injected with DNA copies of HDV-RNA and RNA itself [13,14,15].

Other experiments have shown that, after transfection with an HDV construct, viral replication is also supported by human hepatocellular carcinoma cell lines (Alexander cells) that contain HBV integrants, but no HBV replication intermediates, indicating that, even in the absence of the full HBsAg protein, HDV may be rescued to productive infection by HBV integrants [16]. HDV-RNA synthesis persisted in NTCP-transduced Hep-G2 cells and dividing primary human hepatocytes, despite the inhibition of the extracellular spreading of HBV by the HBsAg blocking agent Myrcludex [17]; thus, human cell divisions allow HDV to be amplified and survive liver regeneration. In humans, the HBV-independent replication of HDV has occurred for a short time exclusively in the context of liver transplantation [18].

### 2.3. Transmission of the HDV through the Envelope of Viruses Other Than HBV

It was recently shown that HDV can be coated and transmitted through the envelope of viruses different from HBV [19]. The HDV ribonucleoprotein was packaged in vitro in the envelope glycoproteins from several viruses, including vesiculovirus, arenavirus, metapneumovirus, and flaviviridae (HCV E1 and E2, dengue, or west Nile virus); infected cells released HDV particles into the extracellular milieu, which then entered cells that expressed the appropriate receptors. In addition, HCV could keep spreading HDV infection in the liver of co-infected humanized mice for several months; same as for the enveloping with HBsAg, the farnesylation of the HDV ribonucleoprotein was required to assemble particles with the coat of different viruses.

Given the worldwide endemicity of HCV, the obvious question is whether HDV-RNA could be transmitted to humans through the envelope glycoproteins of HCV. In a study of 2123 plasma samples positive for anti-HCV antibody, a prevalence of anti-HD of 1.9% has been reported; of the 41 anti-HD-positive samples, 27 (65.9%) were positive for anti-HBc. However, no anti-HDV-positive sample was positive for HDV-RNA [20]. Another study of 323 subjects infected with HCV and negative for HBsAg failed to identify HDV-RNA in all patients; 8 out of 323 subjects had anti-HD, but all were positive for anti-HBc as a sign of past HBV infections [21]. Finally, a recent study identified one HDV-RNA-positive case in a cohort of 160 HCV-RNA-positive Venezuelan subjects; though the HDV-RNA positive subject had no serological sign of previous HBV exposure, we cannot exclude an occult HBV infection owing to the lack of the corresponding liver tissue samples [22]. One may further speculate whether HDV, disguised as a satellite of viruses different from HBV, could cause human disease other than hepatitis D.

### 2.4. Origin of the HDV

Attention focused initially on an origin of HDV from plant viroids, subviral plant agents that have a very small genomic size compatible with that of HDV [23]; like HDV, they parasite the transcription machinery of the infected plant. A first hypothesis was that a smaller-sized plant satellite RNA that underwent recombination and acquired a cellular RNA encoding HD-Ag was the source of HDV, and later coevolved with HBV, which determined the hepatotropism.

A second hypothesis was that HDV emerged from aberrant HBV-RNA splicing [24]. Following the recognition that a large number of cellular RNAs are converted into circular species that are resistant to host nucleases, it was proposed that, in hepatocytes infected with HBV, some HBV-RNAs may be processed to form circular molecules that in turn can be replicated by the host machinery and assembled using the envelope proteins of HBV; during replication, the RNA could undergo several nucleotide changes, evolving into a different infectious agent such as HDV [25].

Recently, hepatitis D relatives (HDV-like) have been discovered by metagenomics analyses in different species of vertebrates and invertebrates, from birds to reptiles, fishes, and termites [26,27]. Phylogenetic analysis showed that, compared with avian, reptile, fish, or amphibian HDV-like viruses, mammalian delta viruses are closer to a common HDV ancestor [28].

The new findings suggest that HDV-like viruses not dependent on HBV-related hepadnaviruses have probably existed throughout the entire evolution of the metazoan [28,29].

## 3. The Contemporary Epidemiology of HDV

### 3.1. The Antibody to HDV and the Hallmark of Infection

HDV is transmitted by co-infection with HBV or by super-infection on a pre-existing HBsAg-positive state. In both cases, the diagnosis is established by testing for the antibody to HDV (anti-HD) that represents the standard serologic marker for clinical and epidemiologic screening; the measurement of serum HDV-RNA by reverse transcription PCR confirms an active infection [30].

Self-limited acute coinfections are now very rare in Italy; their incidence diminished from 3.2 cases per 1 million inhabitants in 1987 to 0.04 cases in 2019 [31]. In contrast, the superinfection process supports the endemicity of HDV, maintaining the transmission of the virus from HDV-infected to HDV-susceptible carriers; carriers of HBsAg epitomize both the reservoir and the victims of the infection [32].

**Figure 3:**
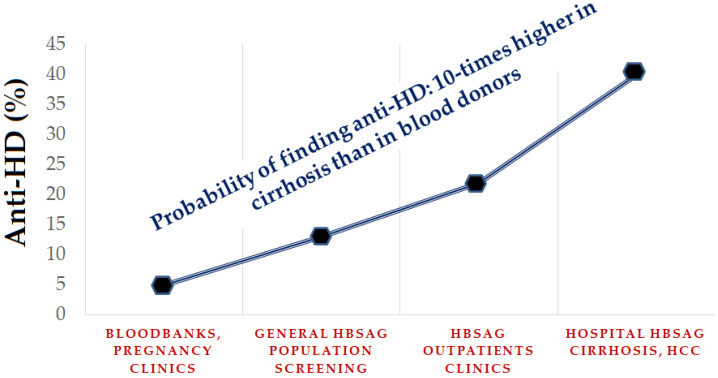
Prevalence of anti-HD in HBsAg carriers at low and high risk of HDV recruited at different sites (extrapolated from data in Italy in the 1980s). Abbreviations: anti-HD, antibodies to hepatitis D; HCC, hepatocellular carcinoma.

The anti-HD is a marker of exposure to HDV and its clinical interpretation must be correlated with the medical status of the HBsAg carrier recruited for the examination [33]. All clinical studies have shown that HDV is pathogenic, most often causing progressive liver disease. Within 5 to 10 years of infection, approximately 70% of cases develop liver cirrhosis; the risk of progression is threefold higher in HDV/HBsAg co-infected as compared with HBV mono-infected patients [34]. Hence, the chances of detecting anti-HD throughout the clinical spectrum of HBsAg-positive liver infections is five- to tenfold lower in asymptomatic patients than in patients with cirrhosis (Table 1) (Figure 3) [35,36,37,38,39,40].

In carriers with liver disease at high risk of HDV, the antibody is not only a marker of exposure to the virus, but also a surrogate marker of HDV replication; when present at serum dilutions 1:1000 or above, it is virtually diagnostic of productive infection and chronic hepatitis D (CHD). In asymptomatic HBsAg carriers at low risk of HDV, the antibody represents most often the serological scar of a past resolved infection. Nevertheless, some of these carriers may have an unrecognized HDV infection: serum HDV-RNA was positive in 23% of 61 asymptomatic blood donors collected from 1997 to 2011 in the French National Database of Blood Donors [41], and in 15% of 13 anti-HD positive blood donors recruited in Turkey in the 2010s [39]. It is thus rational to test for serum HDV-RNA as all the HBsAg carriers who have no overt liver disease, as some could harbour a latent HDV infection. The optimal screening approach is by reflex testing, i.e., the algorithm that implies the automatic determination of anti-HD in all individuals resulting positive for HBsAg, regardless of clinical status [42].

The clinical context of patients surveyed for anti-HD is also critical in assessing figures of prevalence. Surveying performed for recruiting convenience in HBsAg carriers at low risk of HDV, like in blood banks or social and working communities, provides a rate of infection distinctly lower than in carriers with liver disease recruited in hospital and at hepatology centers [33]. Thus, extrapolating prevalence results to the general population without considering the medical background of the patients, whether at low or high risk of HDV, may be misleading; only the prevalence in HBsAg carriers with liver disease at high risk of HDV provides reliable figures on the true impact of HDV in a country.

### 3.2. The Epidemiological Scenario

With the introduction of vaccination against HBV, the global scenario of HDV has changed in the last decades; HBV vaccination is the most effective method of preventing HDV infections through the depletion of the network of HBsAg carriers.

**Figure 4:**
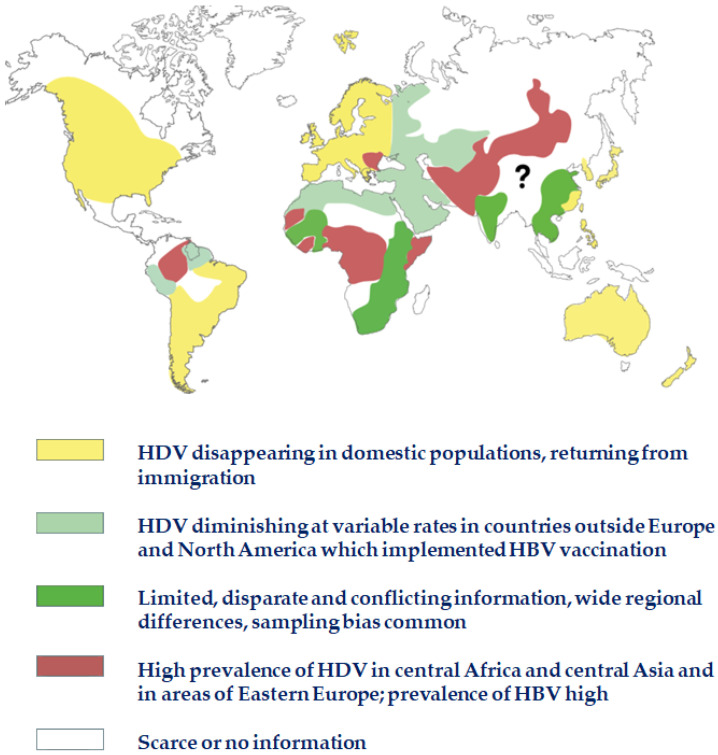
Contemporary HDV prevalence. Abbreviations: HBV, hepatitis B virus; HDV, hepatitis D virus.

HBV vaccine campaigns began in the 1990s in high-income countries; at present, the younger generations are immune to HBV and, by default, to HDV. In subsequent years, HBV vaccination was introduced into many other countries, changing the scenario of hepatitis D in less affluent countries too, in function of the degree of partial protection afforded so far; where HBsAg prevalence is declining, HDV is coming under control, and younger generations are becoming more protected (Figure 4) [33].

In 1991, Italy was the first country to introduce universal HBV vaccination, and has been central to the unfolding of the epidemiological and clinical changes of HDV; the country is thus suited to provide the paradigm of the contemporary scenario of HDV in the industrialized world.

**Figure 5:**
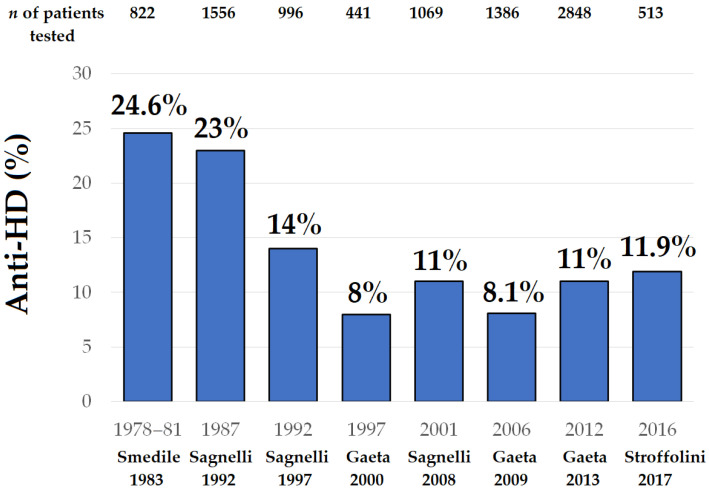
Decline in anti-HD in HBsAg carriers with liver disease in Italy by the end of last century and return of the antibody with migratory fluxes in the last two decades. Abbreviations: anti-HD, antibodies to hepatitis D; *n*, number.

The prevalence of anti-HD in 1983 in Italy was 24% in HBsAg liver disease, with a peak close to 50% in HBsAg(+) cirrhotics in Southern Italy [36]. Since then, the scenario has drastically changed as a result of different and opposite epidemiological trends in native Italians and immigrants. Improvements in socio-sanitarian conditions led in 1992 to a 14.4% decline in HDV in domestic HBsAg(+) carriers (Figure 5) [43], and then to a fall to 6.4% in 2019 with the progress of the vaccination campaign [44]. The younger generations, historically most exposed to the HDV, are by now fully protected; in 2014, only 61 of 513 (11.9%) HBsAg carriers recruited in Italy had anti-HD and only 3% were younger than 30 years (Table 2) [31,45,46]. The vaccine program is ongoing, with the age of subjects immune to HBV/HDV increasing every year, with the perspective that, in native Italians, HDV infection will vanish with the next generation.

However, though extinguishing in the indigenous population, HDV has not run out in the country, but is increasingly reintroduced by immigrants coming from areas where the infection remains endemic [33]. In the period 2001–2009, the prevalence of anti-HD diminished from 7.4 % to 6.4% among Italians, but increased from 12.9% to 26.4% in immigrants [44]; in all high-income countries, a pattern of declining domestic and rising migrant HDV infections is emerging.

**Figure 6:**
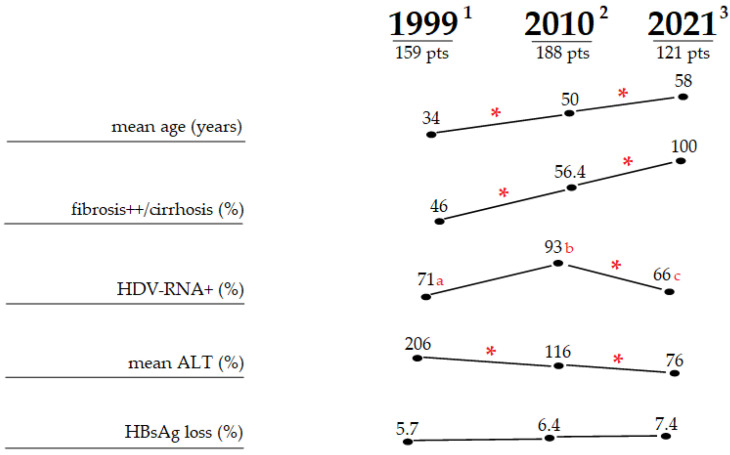
Features of HDV Italians recruited at different time-points. * *p* value < 0.001. ^1^ [48], ^2^ [49] ^3^ [45]. HDV-RNA measured by ^a^ qualitative methods, ^b^ semiquantitative PCR, and ^c^ quantitative PCR. Abbreviations: ALT, alanine aminotransferase; HBsAg, hepatitis B surface antigen; pts, patients.

In Italy, the decline of HDV has been accompanied by changes in the medical profile of HDV disease, documented by four sequential clinical studies every ten years in the last four decades (Figure 6) [45,47,48,49]. Throughout this time, the perception of CHD changed from a severe disease most often predisposing to liver failure to a picture also including long-standing liver disorders. In the last survey reported in 2021 [45], the median age has increased to 58 years and the rate of overt cirrhosis to 71%. The median ALT values and HDV viremia diminished, with about one-quarter of the patients clearing serum HDV-RNA and 7.4% also clearing HBsAg; new and fresh forms of CHD have no longer occurred among native Italians.

The epidemiologic evolution indicates that, in native Italians, CHD survives in a cohort of ageing patients with advanced fibrotic disease of diminished viral and inflammatory activity, which represents the tail of an infection acquired decades ago when HDV was endemic in Italy. This cohort still has an impact on liver transplantation programs. The number of HDV liver transplants performed in Torino from 2010 to 2019 was 130 versus 129 HBV transplants [18], demonstrating an imbalance between the high proportion of HDV transplants and the low weight of HDV infection in the current HBsAg(+) scenario (Table 3).

The different efficacy of therapies in chronic hepatitis B (CHB) and in CHD could explain this paradox; the only available therapy for CHD has remained interferon-alpha (IFN-α), which failed to control HDV infection in a significant proportion of patients, while over 90% of CHB cases have not progressed to terminal disease thanks to the effective antivirals used in the last 20 years. As a consequence, the request for liver transplant in CHB patients drastically diminished. In the last decade, 62.3% of the HDV patients were transplanted for liver failure and 37.7% for hepatocellular carcinoma (HCC), while only 29.5% of HBV patients underwent liver transplant for liver failure and 70.5% for HCC [18].

## 4. Therapy of Chronic Hepatitis D

Treatment of CHD has so far remained an unsolved medical need. The only therapy approved by professional societies is pegylated interferon-IFNα (Peg-IFNα), which, however, has limited efficacy [50].

The unique virology of HDV is challenging [5]. Compared with ordinary viruses, HDV is too small to encode the viral proteins responsible for an autonomous replication. Consequently, HDV depends on the synthetic machinery of the infected hepatocytes for replication, duplicating the viral genome by subverting DNA-dependent RNA polymerases to copy viral RNA [51]; thus, HDV cannot be treated by conventional antivirals targeted against virus-coded proteins [52].

A further issue is represented by the high infectivity of HDV on the background of a pre-existing HBV infection; an HDV-containing serum could transmit infection up to a 10^−11^ dilution, as demonstrated by end-titration studies in HBsAg-susceptible chimpanzees [53]. Therefore, HBsAg persistent in patients who reached a sustained viral response (SVR) can post-therapy rescue HDV that may be present at low levels in the liver, but is undetectable in serum by current available assays [54]. A controversial issue is the end-point of CHD therapy; so far, the criterion for final efficacy has been the clearance of serum HDV-RNA (SVR), representing the best goal that can be achieved in actual practice. Recently, however, a ≥2 Log reduction of serum HDV-RNA from baseline, initially proposed as an interim treatment efficacy endpoint in CHD clinical trials, has been used as an off-treatment endpoint of therapeutic efficacy [55].

Current therapeutic strategies aim to deprive the HDV of the HBsAg functions critical to its life cycle. Currently, three therapeutic venues are currently in evaluation, including drugs that interfere with HBsAg binding to the NTPC to prevent the access of HDV into the hepatocytes [56], drugs that interfere with l-HD-Ag farnesylation to disrupt HDV assembly [57], and drugs that inhibit the synthesis of HBsAg subviral particles to prevent HDV export in the circulation [58].

### 4.1. Nucleic Acid Polymers

Nucleic acid polymers (NAPs) are credited to prevent subviral HBsAg particles from assembling and releasing into the bloodstream. In an open-label, phase 2 trial, NAP REP 2139-Ca was administered as monotherapy to 12 patients with CHD for 15 weeks, followed by 15 weeks of add-on Peg-IFN and then 33 weeks of Peg-IFN monotherapy; overall, 7 patients achieved undetectable HDV-RNA and 4 patients lost HBsAg [59]. These results were maintained after 3.5 years of follow-up [60]. These excellent preliminary data in a small series need to be confirmed in a larger series.

### 4.2. The Farnesyl-Transferase Inhibitor Lonafarnib

In a pilot study, the oral administration of farnesylation inhibitor Lonafarnib (LNF) decreased serum HDV-RNA levels in a dose-dependent manner, but several gastrointestinal side effects were registered [61]. In subsequent studies, LNF has been given in combination with Ritonavir to allow the use of lower doses of LNF without reducing its antiviral activity. In the LOWR-2 study, 5 out of 13 patients given LNF at a dose of 50 mg in combination with Ritonavir at a dose of 100 mg for 24 weeks cleared HDV-RNA [62]. In the LNF + Ritonavir + PEG-IFN lambda 180 µg/week (LIFT-HDV) study, 11 of 26 patients treated for 24 weeks cleared serum HDV-RNA [63]; IFN lambda is credited to be as effective as IFNα, but with less side effects.

### 4.3. Bulevirtide

Bulevirtide (BLV), previously Myrcludex B, is a myristolated synthetic lipopeptide that competitively binds the NTCP, blocking the engagement of HBsAg of HDV, thus preventing the virus entry into yet uninfected hepatocytes [64]. The aim is to eliminate the HDV-infected hepatocytes and recolonize the liver with HDV-free regenerating cells. It is administered subcutaneously daily and well tolerated; in July 2020, it was granted by the European Medicines Agency conditional marketing authorization at a daily dose of 2 mg under the trade name Hepcludex [65].

In a multicenter, open-label, phase 2b clinical trial (MYR 202), BLV was administered at 2, 5, and 10 mg daily in combination with tenofovir alafenamide fumarate (TDF); all patients started TDF at least 12 weeks prior to starting BLV and continued TDF for 24 weeks after BLV [66]. At the end of BLV treatment, HDV-RNA decreased by ≥2 Log or became undetectable in 46%, 47%, and 77% of the patients treated with 2, 5, and 10 mg BLV, respectively. Normalization of ALT was achieved by 43%, 50%, and 40% of the patients, respectively. To note, most patients experienced an HDV-RNA relapse at 12 weeks after BLV cessation.

In the MYR 203 study [67,68], 90 patients were randomized into 6 groups of 15 patients each and treated for 48 weeks. At week 24 of therapy, 8 (53%), 4 (27%), and 1 (7%) of the patients treated with 2, 5, or 10 mg BLV in combination with Peg-IFN, respectively, had undetectable HDV-RNA. Only 1 (7%) patient with BLV 2 mg given in the arm in monotherapy and 3 (33%) patients with BLV 10 mg given in the arm in combination with TDF achieved undetectable HDV-RNA after 24 weeks of post-therapy follow-up, while none of the patients treated with Peg-IFN alone cleared HDV-RNA. Four out of 15 patients treated with the combination Peg-IFN with BLV 2 mg lost HBsAg.

Two long-term studies have been implemented, one of finite therapy with BLV in combination with Peg-IFN (MYR-204; phase 2b), and the other of indefinite therapy with BLV alone (MYR-301; phase 3). Data from both studies were reported at an interim of 24 weeks. In the MYR-204 study, where the primary endpoint was undetectable HDV-RNA, patients were treated with Peg-IFN alone (*n* = 25), BLV 2 mg plus Peg-IFN (*n* = 50), BLV 10 mg plus Peg-IFN (*n* = 50), and BLV 10 mg (*n* = 50) [69]. At week 24 of therapy, 13, 24, 34, and 4 patients achieved undetectable serum HDV-RNA, and 13%, 30%, 24%, and 64% normalized the ALT, respectively. In the MYR-301 study, patients were treated with BLV 2 mg (*n* = 49), BLV 10 mg (*n* = 50), and left untreated (*n* = 51); the primary endpoint was the normalization of ALT in combination with HDV-RNA undetectability or ≥2 Log IU/mL decrease from baseline [70]. The endpoint was achieved by 6%, 53%, 38%, and 6% patients, respectively.

In a compassionate French study [71], patients treated with BLV 2 mg alone (*n* = 77) or BLV 2 mg combined to Peg-IFN (*n* = 68) were included in a per-protocol analysis. At 12 months, undetectable HDV-RNA was achieved by 39% of the former and 85% of the latter. In addition, 48.8% of the patients treated with BLV alone and 36.4% of the patients treated with combination therapy normalized ALT serum values. The virologic results of the combination therapy are outstanding, but need to be validated by further prospective randomized study including patients with comparable demographic and clinical characteristics.

Finally, in a real-world study, 23 patients started BLV monotherapy and 10 (45%) of them were classified as responders (HDV-RNA decrease >2 Log or undetectable) at week 24. ALT normalized in 14 (65%) patients. The addition of peg-IFN in 8 patients further induced an HDV-RNA decrease. Notably, portal pressure decreased in 2 out of 5 (40%) patients undergoing repeated measurement under BLV therapy [72].

In summary, BLV and LNF as monotherapy show partial efficacy in reducing HDV-RNA in serum. Combined with Peg-IFN, they provide a better therapeutic effect, and this combination appears to represent the best therapy for patients with CHD that can tolerate Peg-IFN. For patients who cannot tolerate Peg-IFN, long-term BLV monotherapy may offer an alternative, despite being less active against HDV than the combinations, resulting in good biochemical responses and tolerability.

## Figures and Tables

**Table 1:** Different prevalence of anti-HD in HBsAg blood donors and HBsAg cirrhotics.

	Italy, 1985	Iran	Turkey	Uzbekistan
Donors	5% [35]	2% [37]	7% [38]	8 *
Cirrhotics	51% [36]	66% [37]	61.4% [39]	>80% [40]

* Musabaev E, personal communication.

**Table 2:** HDV infection in 2021 in high-income countries: the paradigm of Italy.

HDV vanishing in the domestic population, accounting for about 5000 patients with fibrotic/cirrhotic disease (mean age of 58 years in 2020) [45]
In 2017, the proportion of HDV-positive native Italians younger than 30 years = 3% [46]
Acute HDV incidence per 1-million population; from 3.2 cases in 1987 to 0.04 in 2019 [31]

**Table 3:** HBsAg liver transplants from 2010 to 2019 in Torino, Italy.

	Total	HBV	HDV
HBsAg patients, *n* (%)	259	129 (50%)	130 (50%)
Indication for transplant (%) [18]			
Liver failure		25.9%	62%
HCC		70.5%	38%

Abbreviations: HBsAg, hepatitis B surface antigen; HBV, hepatitis B virus; HCC, hepatocellular carcinoma; HDV, hepatitis D virus; *n*, number.

## Data Availability

Not applicable.
